# Reduced Activity of Mutant Calcium-Dependent Protein Kinase 1 Is Compensated in *Plasmodium falciparum* through the Action of Protein Kinase G

**DOI:** 10.1128/mBio.02011-16

**Published:** 2016-12-06

**Authors:** Abhisheka Bansal, Kayode K. Ojo, Jianbing Mu, Dustin J. Maly, Wesley C. Van Voorhis, Louis H. Miller

**Affiliations:** a Laboratory of Malaria and Vector Research, National Institute of Allergy and Infectious Diseases, National Institutes of Health, Rockville, Maryland, USA; b Division of Allergy and Infectious Diseases, Department of Medicine, and Center for Emerging and Re-emerging Infectious Diseases, University of Washington, Seattle, Washington, USA; c Departments of Biochemistry and Chemistry, University of Washington, Seattle, Washington, USA

## Abstract

We used a sensitization approach that involves replacement of the gatekeeper residue in a protein kinase with one with a different side chain. The activity of the enzyme with a bulky gatekeeper residue, such as methionine, cannot be inhibited using bumped kinase inhibitors (BKIs). Here, we have used this approach to study *Plasmodium falciparum* calcium-dependent protein kinase 1 (*Pf*CDPK1). The methionine gatekeeper substitution, T145M, although it led to a 47% reduction in transphosphorylation, was successfully introduced into the CDPK1 locus using clustered regularly interspaced short palindromic repeat (CRISPR)/Cas9. As methionine is a bulky residue, BKI 1294 had a 10-fold-greater effect *in vitro* on the wild-type enzyme than on the methionine mutant. However, in contrast to *in vitro* data with recombinant enzymes, BKI 1294 had a slightly greater inhibition of the growth of CDPK1 T145M parasites than the wild type. Moreover, the CDPK1 T145M parasites were more sensitive to the action of compound 2 (C2), a specific inhibitor of protein kinase G (PKG). These results suggest that a reduction in the activity of CDPK1 due to methionine substitution at the gatekeeper position is compensated through the direct action of PKG or of another kinase under the regulation of PKG. The transcript levels of CDPK5 and CDPK6 were significantly upregulated in the CDPK1 T145M parasites. The increase in CDPK6 or some other kinase may compensate for decrease in CDPK1 activity during invasion. This study suggests that targeting two kinases may be more effective in chemotherapy to treat malaria so as not to select for mutations in one of the enzymes.

## IMPORTANCE

Protein kinases of *Plasmodium falciparum* are being actively pursued as drug targets to treat malaria. However, compensatory mechanisms may reverse the drug activity against a kinase. In this study, we show that replacement of the wild-type threonine gatekeeper residue with methionine reduces the transphosphorylation activity of CDPK1. Mutant parasites with methionine gatekeeper residue compensate the reduced activity of CDPK1 through the action of PKG possibly by upregulation of CDPK6 or some other kinase. This study highlights that targeting one enzyme may lead to changes in transcript expression of other kinases that compensate for its function and may select for mutants that are less dependent on the target enzyme activity. Thus, inhibiting two kinases is a better strategy to protect the antimalarial activity of each, similar to artemisinin combination therapy or malarone (atovaquone and proguanil).

## INTRODUCTION

Potential resistance against the front-line drug artemisinin warrants the search for new drug targets (1–3). Protein kinases have been extensively explored and exploited as drug targets to treat various human diseases (4). In the last decade, *Plasmodium* kinases have been investigated as potential drug targets to treat malaria (5). Since kinases play indispensable roles at various stages of the *Plasmodium falciparum* life cycle, studying their function by gene knockout techniques may not be feasible (6). An elegant method to study mammalian kinase-specific biochemical functions through chemical genetics, known as the sensitization strategy, was developed almost 2 decades ago (7). The strategy involves engineering a unique pocket in the active site of a target kinase at a position called the gatekeeper position. The gatekeeper residue is adjacent to the ATP binding site and mainly consists of bulky amino acid residues such as methionine, isoleucine, or leucine in mammalian kinases. Replacement of the bulky residue in the target kinase with a smaller residue such as alanine, glycine, or serine renders the enzyme sensitive toward a particular group of inhibitors known as bumped kinase inhibitors (BKIs). This strategy could also be utilized in the reverse order by replacing a smaller gatekeeper residue with a bulky residue, thereby making the enzyme resistant to treatment with BKIs.

Often, however, the replacement of the gatekeeper residue has a dramatic effect on the activity of the target kinase, which could lead to a reduction or complete abrogation of the kinase activity (8, 9). The gatekeeper substitution could have a distinct effect on the activity of the enzyme in terms of autophosphorylation and transphosphorylation of the substrate. The failure of transphosphorylation due to gatekeeper substitutions would probably indicate that the mutation in an essential protein kinase will be lethal to the parasite. Therefore, it becomes imperative to evaluate the effect of gatekeeper substitutions on the kinase activity of the target enzyme by methods that can differentiate between the two events of phosphorylation before proceeding to the allelic exchange in the parasite.

**FIG 1:**
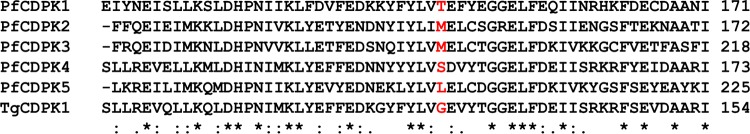
Protein sequence alignment highlighting gatekeeper residues in *Plasmodium falciparum* CDPKs and *Toxoplasma gondii* CDPK1. The gatekeeper residues shown in red are based on the glycine residue at position 128 of *Tg*CDPK1 (17). Methionine in *Pf*CDPK2 and -3 (PlasmoDB accession numbers PF3D7_0610600 and PF3D7_0310100, respectively) and leucine in *Pf*CDPK5 (PlasmoDB accession no. PF3D7_1337800) are large gatekeeper residues compared to threonine in *Pf*CDPK1 (PlasmoDB accession no. PF3D7_0217500). *Tg*CDPK1 and *Pf*CDPK4 (PlasmoDB accession no. PF3D7_0717500) have glycine and serine, respectively, which are smaller than threonine.

In this study, we have investigated the effect of gatekeeper substitution on the activity of *Plasmodium falciparum* calcium-dependent protein kinase 1 (*Pf*CDPK1). The CDPK family in *P. falciparum* is composed of seven members. CDPKs have an N-terminal kinase domain and a C-terminal calmodulin-like domain with various numbers of EF-hands. Binding of calcium ions with the EF-hands modulates the protein conformation with concomitant induction of kinase activity. The gatekeeper position in *Pf*CDPK2, -3, and -5 is constituted by bulky methionine, methionine, and leucine residues, respectively, and *Pf*CDPK4 has a small gatekeeper, serine (Fig. 1). The gatekeeper position in wild-type (WT) *Pf*CDPK1 is constituted by threonine (T145), which falls between a small and a bulky residue. *Pf*CDPK1 has been shown to play an important role in different critical processes in the asexual proliferation of the parasite within erythrocytes (10–13). Attempts to knock out *Pf*CDPK1 from the asexual stage in the WT parasite have not been successful, indicating that the gene may have an essential role in the WT parasite (6, 11). However, *Pf*CDPK4 was successfully disrupted in the blood-stage parasite, suggesting a redundant role or none for asexual proliferation of the parasite (6, 11).

We have selected five different residues to study the effect of the gatekeeper substitution on the activity of recombinant *Pf*CDPK1. Glycine, alanine, and serine, which are smaller, and methionine and tyrosine, which are bulky compared to the threonine residue in the wild-type *Pf*CDPK1, were introduced in the gatekeeper position by site-directed mutagenesis and expressed as N-terminal glutathione *S*-transferase (GST)-tagged chimeric proteins in *Escherichia coli* along with the wild type. We have used a “semisynthetic epitope” tagging approach, a nonradioactive Western blotting method, to detect autophosphorylation and transphosphorylation potential of all the recombinant protein kinases using ATPγS (14).

We show here that replacement of a gatekeeper threonine with a smaller gatekeeper residue, serine, alanine, or glycine, dramatically affects the kinase activity of *Pf*CDPK1 with complete abrogation of transthiophosphorylation of histone H3, although the proteins retain autophosphorylation activity. Attempts to transform *P. falciparum* with the serine at the gatekeeper position were unsuccessful, likely due to toxicity for the parasite. On the other hand, the methionine mutation of the CDPK1 gatekeeper retains 53% transphosphorylation activity. We have also generated a transgenic parasite strain with a T145M substitution in the *Pf*CDPK1 gene using the latest gene-editing technique, the clustered regularly interspaced short palindromic repeat (CRISPR) and CRISPR-associated protein (Cas) system (CRISPR/Cas9) (15). Here, we show that reduction in the kinase activity of CDPK1 is compensated in the transgenic parasites by protein kinase G (PKG) or increased expression of a kinase regulated by PKG. Additionally, we found that CDPK5 transcript expression was upregulated in the mutant parasites. A similar study with knockout of Pfmap-1 in *P. falciparum* showed an increase of the Pfmap-2 protein level (16).

## RESULTS

### Recombinant *Pf*CDPK1 WT and gatekeeper mutants are bona fide calcium-dependent kinases.

**FIG 2:**
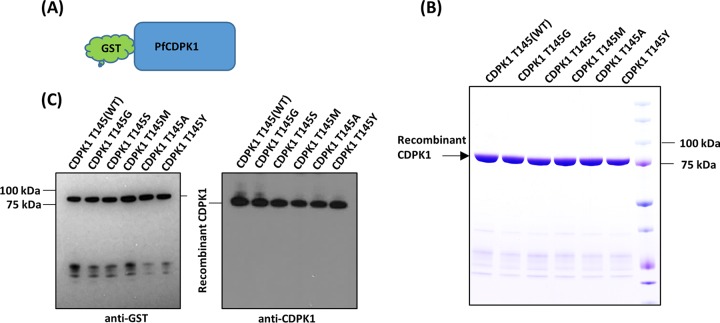
Characterization of purified recombinant full-length *Pf*CDPK1 wild-type (WT) and gatekeeper mutant proteins. (A) Full-length CDPK1 WT and five mutant proteins with different substitutions at the gatekeeper position (residue 145) were expressed in *E. coli* as N-terminal glutathione *S*-transferase (GST) fusion proteins as depicted in the cartoon. (B) The chimeric fusion proteins were purified by affinity chromatography, exploiting the interaction of glutathione with the GST, and separated by 4 to 12% SDS-PAGE. All the proteins migrated to the predicted molecular mass of ~87 kDa as shown in the Coomassie blue-stained polyacrylamide gel. (C) SDS-PAGE-separated proteins were transferred to a PVDF membrane and processed for Western blot analysis. The membranes probed using anti-GST and anti-CDPK1 antibodies confirmed the identity of the purified recombinant proteins. CDPK1 wild type (WT) and mutants with gatekeeper replacements of glycine (G), serine (S), methionine (M), alanine (A), or tyrosine (Y) are shown.

**FIG 3:**
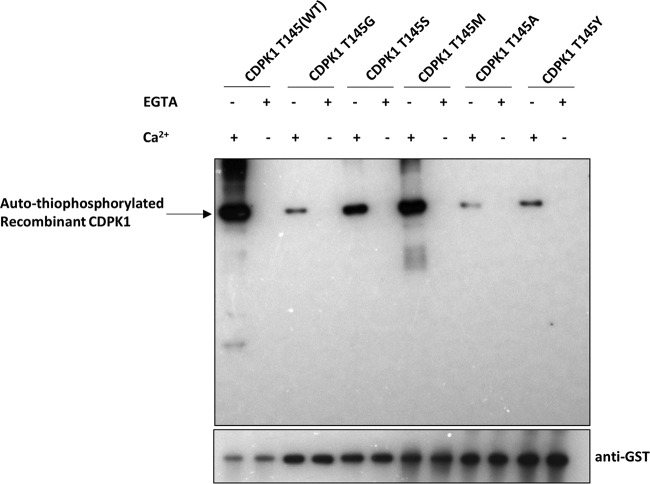
The kinase activity of recombinant CDPK1 WT and gatekeeper mutant proteins is dependent on the presence of Ca^2+^ ions. The Western blot shows the autophosphorylation of recombinant CDPK1 WT and the gatekeeper mutant proteins with a specific antibody that detects the thiophosphorylated substrates in the presence of calcium chloride (+ Ca^2+^) and no autophosphorylation in the presence of EGTA. The lower panel shows the same blot stripped and reprobed with anti-GST antibodies. WT denotes CDPK1 wild type with threonine (T) at gatekeeper position 145. T145G, T145S, T145M, T145A, and T145Y denote CDPK1 mutant proteins with glycine, serine, methionine, alanine, or tyrosine, respectively, at the gatekeeper position.

Sixty amino acid residues in the kinase domain of *Pf*CDPK1 to *Pf*CDPK5 were aligned with *Toxoplasma gondii* CDPK1 (*Tg*CDPK1) as a template. The gatekeeper residue is the amino acid where ATP enters the active site of the kinase. The gatekeeper residues of *Pf*CDPK1 to *Pf*CDPK5 are aligned with G128 of *Tg*CDPK1 (Fig. 1) (17). *Pf*CDPK1 wild type (WT) and five different mutants with alanine, glycine, serine, methionine, or tyrosine at the gatekeeper position were expressed as full-length proteins with an N-terminal glutathione *S*-transferase (GST) tag (Fig. 2A). The gatekeeper mutants were made to study the effect of these substitutions on the kinase activity of *Pf*CDPK1. The affinity-purified recombinant chimeric proteins were of the expected molecular mass of ~87 kDa (Fig. 2B). All the proteins were detected with anti-GST and anti-*Pf*CDPK1 sera, indicative of the chimeric protein identity (Fig. 2C). The *in vitro* kinase assay was set up as described in Materials and Methods to check the effect of calcium on the activity of recombinant CDPK1 WT and the gatekeeper mutants. In the presence of 2 mM calcium chloride, all the kinases were active as detected by autothiophosphorylation of CDPK1, although the glycine, alanine, and tyrosine mutants showed highly diminished levels compared to the WT (Fig. 3). The presence of 2 mM EGTA, with no added Ca^2+^, completely abolished the activity of the recombinant kinases (Fig. 3). Thus, mutations in the gatekeeper residue retained calcium-dependent kinase activity.

### Gatekeeper substitutions in CDPK1 led to reduction or elimination of transphosphorylation activity.

**FIG 4:**
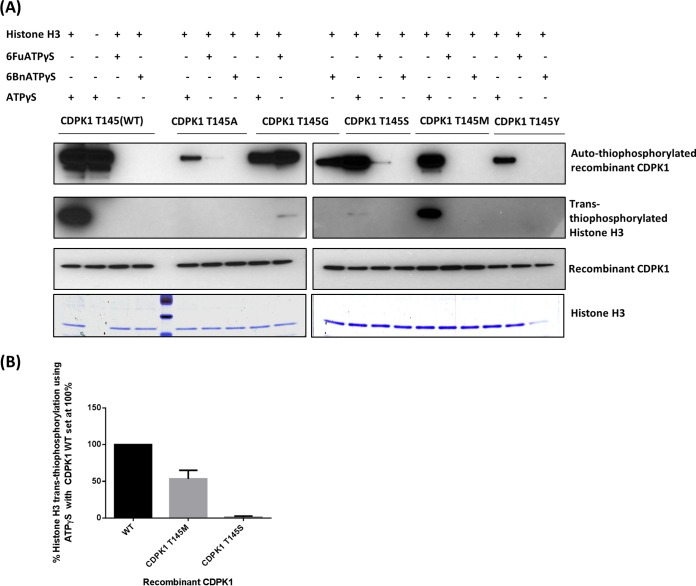
Substitutions at the gatekeeper position of CDPK1 decrease or eliminate the transphosphorylation of histone H3. (A) Western blot shows the autothiophosphorylation of CDPK1 and transthiophosphorylation of histone H3 with WT and gatekeeper mutants. Gatekeeper substitutions with alanine (CDPK1 T145A), glycine (CDPK1 T145G), and tyrosine (CDPK1 T145Y) completely abrogate the transphosphorylation of the mutant enzyme. (B) With the serine (CDPK1 T145S) gatekeeper, the transthiophosphorylation of histone H3 was 98% reduced. The methionine (CDPK1 T145M) gatekeeper mutant retains 53% transphosphorylation activity and best mimics the wild-type enzyme compared to other mutant enzymes. Transthiophosphorylation of histone H3 has been quantified with ImageJ software (version 1.49; National Institutes of Health http://imagej.nih.gov/ij/), and the values of percent histone H3 thiophosphorylation with ATPγS normalized with *Pf*CDPK1 have been plotted for WT, T145S, and T145M. The standard deviation from two experiments for WT, T145S, and T145M is shown.

Gatekeeper mutations were evaluated *in vitro* to determine the effect on autophosphorylation (phosphorylation of the CDPK1 itself) and transphosphorylation (the transfer of phosphate to histone H3) of the recombinant enzymes before attempting to introduce the mutation into the parasite. Gatekeeper replacement with alanine, glycine, or tyrosine drastically reduced the autophosphorylation of recombinant CDPK1 as evident from autothiophosphorylation of the protein (Fig. 3 and 4); in addition, no transphosphorylation was observed (Fig. 4). The absence of transphosphorylation indicates that the gatekeeper mutation may be lethal for the parasite. The serine residue at the gatekeeper position also led to a reduction in autophosphorylation (Fig. 3 and 4), and transphosphorylation of histone H3 was greatly reduced (Fig. 4A and B). Replacement of the gatekeeper residue with methionine (CDPK1 T145M) led to a 47% reduction in transthiophosphorylation of histone H3 (Fig. 4B).

ATP analogs with bulky substitution at the 6-amino group of the adenine ring (6FuATPγS and 6BnATPγS) can be utilized only by a kinase with a small gatekeeper residue (such as glycine, alanine, or serine). The ATP analogs have been used to specifically phosphorylate downstream targets of enzymes with a small gatekeeper residue since the bulky substitutions on ATP analogs can be accommodated only in mutant enzymes with small gatekeepers. The glycine (the smallest residue) gatekeeper mutant of CDPK1 utilized bulky ATP analogs as revealed by autothiophosphorylation and to a small extent for transthiophosphorylation of histone H3 with 6FuATPγS (Fig. 4A). However, the enzymes containing large gatekeepers, such as methionine, tyrosine, or threonine in the wild-type enzyme, were not labeled by the bulky ATP analogs (Fig. 4A). This result shows that the threonine gatekeeper in the wild-type CDPK1 is sufficiently large to block the entry of the bulky ATP analogs.

### Generation of a *Pf*CDPK1 methionine gatekeeper transgenic parasite.

**FIG 5:**
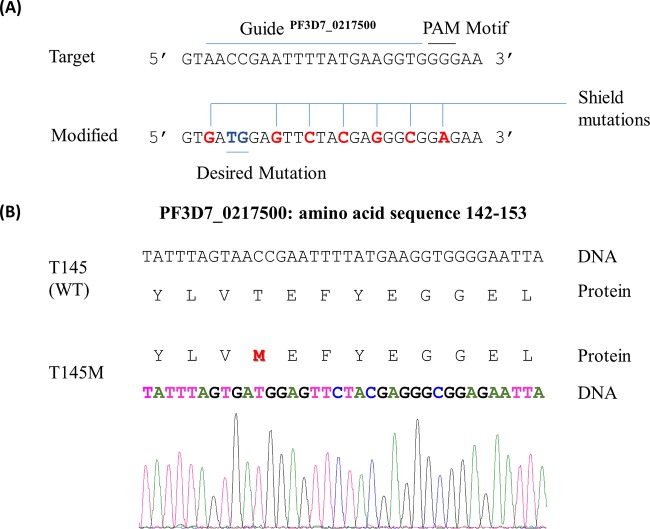
Generation of *Pf*CDPK1 T145M transgenic parasites. Diagrammatic representation of the marker-free DNA-editing strategy using CRISPR/Cas9 to introduce the methionine gatekeeper in endogenous *Pf*CDPK1. (A) The gene sequence of CDPK1 (PlasmoDB accession no. PF3D7_0217500) from nucleotides 424 to 459, highlighting the guide region (20 nucleotides) and the protospacer adjacent motif (PAM), is shown for the WT (target) and the methionine gatekeeper mutant (modified) parasite. The desired mutation (ACC to ATG) resulting in T145M substitution is highlighted in blue, and the shield mutations are shown in red. The shield mutations prevent recutting of the modified locus (GAG) and complementary base pairing with the guide RNA after the incorporation of the desired substitution. (B) The amino acid sequences (residues 142 to 153) of the wild type (T145) and methionine gatekeeper mutant (T145M) are shown with the corresponding DNA sequence. The methionine gatekeeper residue in the T145M mutant has been highlighted in red. The DNA sequence of the methionine gatekeeper mutant was verified by sequencing the gene. The chromatogram corresponding to nucleotides 424 to 459, coding for amino acids 142 to 153, is shown.

The *in vitro* kinase activity data show that the T145M substitution best mimics the activity of the wild type among all the other mutations tested; the serine residue drastically reduces the transphosphorylation potential of the mutant enzyme. We made use of the recent gene-editing technique CRISPR/Cas9 to introduce the methionine or serine gatekeeper residue in the CDPK1 locus. We failed to introduce serine at the gatekeeper position in the endogenous CDPK1 locus since the serine residue drastically reduces the kinase activity of CDPK1. We successfully generated a transgenic parasite strain with a methionine gatekeeper (Fig. 5). The growth rate of two clones of the T145M transgenic parasite was similar to that of the wild-type parasite as estimated by SYBR green I assay over a period of 7 days (see Fig. S1 in the supplemental material).

### Evaluation of BKIs against the recombinant *Pf*CDPK1 wild type and methionine gatekeeper mutant.

We next screened the recombinant wild-type *Pf*CDPK1 and the methionine gatekeeper mutant against 31 bumped kinase inhibitors (BKIs) (18–21). Twenty-six BKIs showed differential inhibition of the wild type and the methionine gatekeeper mutant (Table 1). Two BKIs, 1613 and 1294, inhibited the wild-type recombinant *Pf*CDPK1 with 50% inhibitory concentrations (IC_50_s) of 0.085 and 0.162 μM, respectively, and an IC_50_ of >2 μM against the methionine gatekeeper mutant (Table 1). Thus, the BKIs had a greater effect on the kinase activity of the threonine-containing wild-type CDPK1 than on CDPK1 T145M. Furthermore, BKI 1294 inhibited the kinase activity of recombinant *Pf*PKG with an IC_50_ of 0.262 μM (Table 2). However, *Pf*PKG activity was not inhibited by BKI 1613 (IC_50_, >2 μM) (Table 2). Thus, *Pf*PKG is inhibited only by BKI 1294.

**TABLE 1:** Differential inhibition of kinase activity of recombinant *Pf*CDPK1 WT and *Pf*CDPK1 T145M with different BKIs^a^

Compound	*Pf*CDPK1 WT		*Pf*CDPK1Met IC_50_ (μM)
	IC_50_ (μM)	SD	
1318	0.468	0.069	>2
1369	0.438	0.137	>2
GP 12	>2		>2
EJDI.063	0.460	0.118	>2
RM-1-114A	>2		>2
RM-1-152A	0.029	0.001	>2
RM-1-167	>2		>2
**1294**	0.162	0.072	>2
RM-1-170	0.363	0.252	>2
RM-1-175	1.048	0.918	>2
RM-1-181	>2		>2
RM-1-132	0.148	0.103	>2
RM-1-186	1.335	0.949	>2
1220	0.912	0.231	>2
1224	0.980	0.384	>2
1244	0.465	1 run	>2
1266	0.426	0.085	>2
1275	1.423	0.717	>2
1432	0.148	0.062	>2
1479	0.392	0.198	>2
NAPP2	0.337	0.267	>2
1510	0.599	0.112	>2
1553	0.350	0.171	>2
1537	0.503	0.002	>2
1567	>2		>2
1568	0.333	0.219	>2
1590	0.122	0.084	>2
1601	0.573	0.079	>2
1610	0.641	0.194	>2
**1613**	0.085	0.047	>2
NM-PP1	1.019	0.520	>2

^a^ The effects of 31 different BKIs on the kinase activity of recombinant *Pf*CDPK1 WT and *Pf*CDPK1 T145M have been tabulated as IC_50_s. Two BKIs, 1613 and 1294, highlighted in bold, were tested for inhibition of growth of the CDPK1 T145M and wild-type parasites. Most of the BKIs inhibited the recombinant *Pf*CDPK1 T145M (IC_50_, 0.029 to 1.423 μM) but did not affect the activity of *Pf*CDPK1 WT. The data are generated from two independent experiments run in triplicate.

**TABLE 2:** Effect of BKIs on kinase activity of recombinant *P. falciparum* protein kinase G^a^

BKI	IC_50_, μM (SD) for *Pf*PKG
**1294**	0.262 (0.041)
1318	1.384 (0.551)
**1613**	>2
1369	>2
Rm-1-132	0.374 (0.095)
1533	>2
1266	1.644 (0.183)

^a^ The effects of seven different BKIs on the kinase activity of recombinant *Pf*PKG have been tabulated as IC_50_s. BKI 1294 inhibited the activity of recombinant *Pf*PKG with an IC_50_ of 0.262 μM, while BKI 1613 did not show any inhibition (IC_50_, >2 μM). BKIs 1294 and 1613, highlighted in bold, were also tested *in vivo* for inhibition of growth of the CDPK1 T145M and wild-type parasites. The data are generated from two independent experiments run in triplicate.

### Increased sensitivity of CDPK1 T145M parasites with BKI 1294.

**FIG 6:**
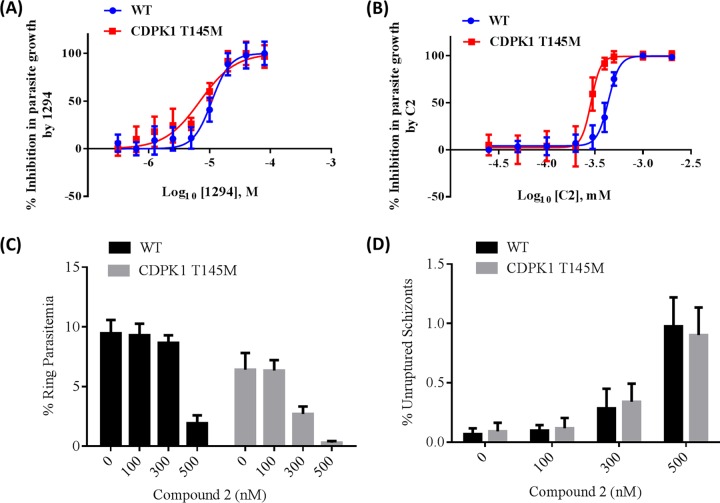
Increased sensitivity of CDPK1 T145M parasites for compound 2 (C2) and bumped kinase inhibitor (BKI) 1294. The effect of BKI 1294 and C2 was evaluated on the asexual proliferation of wild-type and CDPK1 T145M parasites as described in Materials and Methods. (A and B) The figure shows the effect of different concentrations of BKI 1294 (A) and C2 (B) on the growth of wild-type (blue) and CDPK1 T145M (red) parasites. The percent inhibition in parasite growth is plotted against different molar concentrations of BKI 1294 expressed as log_10_[1294] (A). In the case of C2, percent inhibition in parasite growth is plotted against different millimolar concentrations of C2 expressed as log_10_[C2] (B). The figure for 1294 (A) has been generated using three independent experiments, two in duplicate and one in triplicate. The graph for C2 (B) has been generated using data from three independent experiments done in triplicate. All the plots were made using GraphPad Prism 6. (C) Different concentrations of C2 were used to treat wild-type and CDPK1 T145M parasites at 44 to 48 h postinvasion in the schizont stage, and rings were counted 8 h later. The percent ring parasitemia is plotted against different nanomolar concentrations of C2. The graph was plotted using GraphPad Prism 6. The error bars represent standard deviations in two independent experiments done in triplicate. (D) C2 treatment did not show a change in the number of unruptured schizonts in the CDPK1 T145M parasites compared to the WT. The percent unruptured schizonts is plotted against different concentrations of C2 for the CDPK1 T145M and WT parasites. The graph was generated using GraphPad Prism 6, and the error bars represent standard deviations in two independent experiments done in triplicate. WT, wild-type parasite.

Wild-type and CDPK1 T145M parasites were treated with different concentrations of BKI 1294 that showed differential inhibition of WT and CDPK1 T145M recombinant proteins *in vitro* (Table 1). The 50% effective concentrations (EC_50_s) of BKI 1294 in the CDPK1 T145M and wild-type parasites were 6.97 (95% confidence interval, 5.88 to 8.28) and 10.90 (95% confidence interval, 9.76 to 12.16) μM, respectively. The difference in EC_50_ between the CDPK1 T145M and wild-type parasites for BKI 1294 (Fig. 6A), although small, was statistically significant (*P* < 0.02). If BKI 1294 inhibits only *Pf*CDPK1, the *in vitro* studies would predict that the mutant parasites would be more resistant than the wild type. However, BKI 1294 also inhibits recombinant *Pf*PKG (Table 2), and the CDPK1 T145M parasites were more sensitive (not resistant) to the action of BKI 1294 than was the wild type (Fig. 6A), suggesting that *Pf*PKG either directly or through another downstream kinase compensates for the reduced activity of CDPK1 in the mutant parasites. Furthermore, BKI 1613 did not show any difference in inhibition in growth of the two parasites (see Fig. S2 in the supplemental material), likely due to its nonreactivity against *Pf*PKG (Table 2). The EC_50_s for the CDPK1 T145M and the wild-type parasites were 13.1 (95% confidence interval, 10.4 to 16.6) and 14.4 (95% confidence interval, 11.5 to 18.2) μM, respectively.

### Effect of C2: compensation through PKG.

Compound 2 (C2) is a potent and specific inhibitor of protein kinase G (PKG) (22). We used different concentrations of C2 to study its effect on CDPK1 T145M and wild-type parasites. The CDPK1 T145M parasites were more sensitive to C2 treatment than were the wild-type parasites with EC_50_s of 289.5 (95% confidence interval, 276.1 to 303.5) and 437.5 (95% confidence interval, 423.6 to 451.8) nM, respectively (Fig. 6B). The difference in EC_50_ between the CDPK1 T145M and wild-type parasites for C2 was statistically significant (*P* = 0.0002). Furthermore, treatment of late schizont-stage parasites (44 to 48 h postinvasion) with different concentrations of C2 had greater inhibitory effect on the CDPK1 T145M parasites than on the wild type as evaluated by Giemsa smear for the number of ring-infected red blood cells (RBCs) (Fig. 6C). There was a greater decrease in the ring-stage parasites in the CDPK1 T145M parasites than in the wild-type parasites under 300 nM C2 treatment (Fig. 6C; see also Fig. S3B in the supplemental material). The percent unruptured schizonts in the CDPK1 T145M parasites was similar to that of the wild-type parasites under different concentrations of C2 (Fig. 6D). Moreover, the numbers of merozoites in the unruptured schizonts under 300 nM C2 were similar in the CDPK1 T145M and the wild-type parasites (see Fig. S3A). Taken together, the increased sensitivity of CDPK1 T145M parasites to C2, a PKG-specific inhibitor, suggests greater dependence of these parasites on PKG or an alternate pathway involving PKG’s effect on a downstream kinase during invasion of the RBCs.

### Transcript analysis for 11 different kinases by RT-PCR.

**FIG 7:**
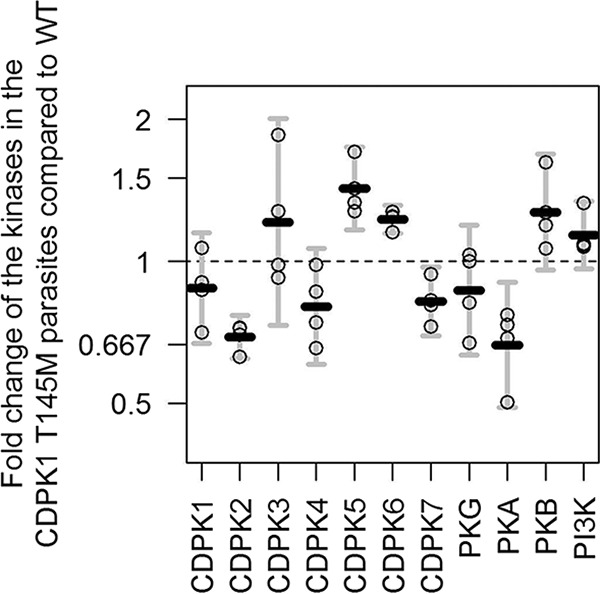
Real-time PCR (RT-PCR) for transcript expression of different kinases in mature schizonts of wild-type and CDPK1 T145M parasites. The difference in the transcript level of 11 different kinases (PlasmoDB numbers in Table 4) was investigated by RT-PCR as described in Materials and Methods. The graph shows the fold change in the expression of the genes in the CDPK1 T145M parasites compared to wild type (set at 1) at the mature schizont stage. Notably, the expression of CDPK5 and CDPK6 is upregulated 1.43- and 1.23-fold, respectively, in the CDPK1 T145M parasites compared to the wild type. The expression of the genes has been normalized with the two housekeeping genes glyceraldehyde-3-phosphate dehydrogenase (GAPDH) and threonine tRNA ligase (ThrRS). The figure has been generated using data from four independent experiments performed in triplicate using R (version 3.3.1) (44). Each circle represent an arithmetic mean of the three replicates on one of the 11 kinases for one of the four experiments. The black solid lines represent the geometric mean of the four points per kinase, and error bars represent 95% confidence intervals on those geometric means, calculated using one-sample t-distribution confidence intervals on the log scale. PI3K, phosphatidylinositol 3-kinase.

The transcript level of 11 different kinases was evaluated by real-time PCR (RT-PCR) in the wild-type and CDPK1 T145M parasites at the mature schizont stage (44 to 48 h postinvasion). These genes were selected based on previous reports of their involvement in egress/invasion of red blood cells (RBCs) or calcium signaling or belonging to the CDPK family. The transcript levels of PKG were found to be similar in the two parasites. However, CDPK5 was significantly upregulated (1.43-fold) in the CDPK1 T145M parasites compared to wild type (Fig. 7 and Table 3). Also, a modest increase was noticed in the transcript level of CDPK6 (Fig. 7 and Table 3). Interestingly, the transcripts of CDPK2, CDPK7, and PKA were found to decrease in the CDPK1 T145M parasites (Fig. 7 and Table 3). All the changes in the expression levels of these genes were found to be statistically significant as tabulated in Table 3.

**TABLE 3:** Two-sided *P* values and geometric mean fold change in expression of CDPK2, CDPK5, CDPK6, CDPK7, and PKA by real-time PCR^a^

Gene (accession no.)	*P* value	Geometric mean fold change for CDPK1 T145M
CDPK2 (PF3D7_0610600)	0.002	0.69
CDPK5 (PF3D7_1337800)	0.011	1.43
CDPK6 (PF3D7_1122800)	0.002	1.23
CDPK7 (PF3D7_1123100)	0.034	0.82
PKA (PF3D7_0934800)	0.024	0.67

^a^ Shown are the *P* values and the geometric mean fold change in the expression of CDPK2, CDPK5, CDPK6, CDPK7, and PKA by real-time PCR in the CDPK1 T145M parasites compared to wild type at the mature schizont stage. The calculations were done in R (version 3.3.1) with two-sided *P* values calculated by a one-sample *t* test. The data are generated from four independent experiments run in triplicate.

## DISCUSSION

The sensitization strategy, a chemical genetics approach, identified the downstream targets of mammalian kinases (23–25), and its importance was also realized for the study of apicomplexan protein kinases (26). Recently, this strategy was used to identify the role of *Toxoplasma gondii* CDPK1 in microneme secretion and host cell invasion (17, 27). Treatment of wild-type *T. gondii* parasites with bumped kinase inhibitors (BKIs) such as 3MBPP1 led to a block in microneme secretion and subsequent invasion of host cells. However, replacement of glycine with methionine (G128M) in the transgenic *T. gondii* parasites conferred resistance to treatment with the BKIs (17, 27). A bulky ATP analog, *N*_6_-furfuryladenosine (kinetin)-5′-*O*-(3-thiotriphosphate) (KTPγS) was subsequently used to label the downstream substrates of *Tg*CDPK1 (28). BKIs inhibited recombinant wild-type *P. falciparum* CDPK4 with serine at the gatekeeper position (29). Moreover, treatment of the gametocytes with BKI 1 inhibited the exflagellation of male gametocytes (29). Replacement of the serine gatekeeper residue with methionine in the transgenic parasites provided resistance to BKI 1 treatment that then failed to inhibit exflagellation (30).

Importantly, replacement of the wild-type gatekeeper residue may cause dramatic changes in the kinase activity of the target enzyme. This could be reflected as reduction or complete abrogation of the kinase activity or change in substrate specificity. The gatekeeper position is more than just a site that allows entry of ATP into the ATP binding pocket. The gatekeeper residue interacts with neighboring residues to maintain the conformation of the ATP binding pocket (8, 9). Substitutions for the wild-type gatekeeper residue could therefore affect the interactions with neighboring residues and may lead to reduction in the affinity for ATP or the target substrate (31). For example, reduced affinity for ATP has been proposed as a reason for low priming-site phosphorylation in the PKCε M486A gatekeeper mutant (31). On the other hand, bulkier, hydrophobic substitutions at the gatekeeper position in tyrosine kinases, such as cellular forms of c-SRC and c-ABL kinases, increased the kinase activity of the mutant enzyme, thereby leading to their oncogenic activation (9). Taking into consideration these observations, we evaluated the effect of gatekeeper substitutions on the activity of *P. falciparum* CDPK1 by *in vitro* assays.

Many kinases undergo autophosphorylation in addition to phosphorylation of their target substrate, transphosphorylation (32). Spectrophotometry and radioactivity-based assays have been routinely used to quantify kinase activity of recombinant enzymes, but these cannot differentiate between autophosphorylation and transphosphorylation. In the present study, we have utilized a “semisynthetic epitope” tagging approach to investigate the effect of gatekeeper mutations on the kinase activity of *Pf*CDPK1 in terms of autophosphorylation and transphosphorylation. Our results suggest that replacement of the wild-type threonine gatekeeper residue of the endogenous *Pf*CDPK1 with alanine, glycine, tyrosine, or serine may be lethal for the parasite as it leads to dramatic reduction in kinase activity of recombinant *Pf*CDPK1. Methionine substitution could be introduced, as that mutant retained 53% transphosphorylation activity. We introduced methionine at the gatekeeper position of endogenous CDPK1 using the marker-free DNA-editing technique, CRISPR/Cas9, but failed to introduce serine. This is in line with the role of CDPK1 in critical processes of erythrocytic asexual proliferation of the parasite, such as egress and invasion (10–13). In contrast, CDPK1 in *Plasmodium berghei* could be knocked out (33). The likelihood of technical difficulty in generating the CDPK1 T145S transgenic parasites is remote since the same guide sequence was used for introducing the methionine gatekeeper substitution in endogenous CDPK1. Moreover, the length and the sequence of the homology arm in the plasmid constructs used for introducing the methionine or serine were the same except for the triplet coding for the amino acid.

Recombinant CDPK1 with a methionine gatekeeper was not inhibited by the BKIs. Therefore, BKIs should not be able to inhibit the growth of mutant parasites with a methionine gatekeeper, CDPK1 T145M. Surprisingly, in contrast to the *in vitro* data with recombinant enzymes, CDPK1 T145M parasites were more sensitive to BKI 1294 treatment than were wild-type parasites. However, BKI 1613 did not show any difference in inhibition of growth of the two parasites. These results suggested that the mutant parasites might deploy compensatory mechanisms for reduced activity of CDPK1 in the CDPK1 T145M parasites that are BKI 1294 sensitive but not affected by BKI 1613. We started exploring the possibility of compensatory mechanisms in CDPK1 T145M parasites given that the methionine gatekeeper mutation led to a decrease in transphosphorylation activity of recombinant CDPK1 and an increase in sensitivity to BKI 1294 of CDPK1 T145M parasites compared to the wild type. Most protein kinase inhibitors, including BKIs 1294 and 1613, are competitive inhibitors with ATP. The cellular concentration of ATP is in the millimolar range compared to micromolar concentrations in the *in vitro* kinase assay. The higher IC_50_s obtained within the parasites with BKIs 1294 and 1613 than in the *in vitro* kinase assay with the recombinant proteins could be due to the high ATP concentration within the parasite. Moreover, a high percentage of target protein kinase activity must be inhibited to detect a phenotype.

*P. falciparum* protein kinase G (*Pf*PKG) plays an indispensable role in egress of merozoites from mature schizonts (22, 34) and subsequent invasion of RBCs (35). *Pf*PKG phosphorylates CDPK1 either directly or through an intermediate kinase in mature schizonts (35). Moreover, the transcript expression profiles of *Pf*PKG and *Pf*CDPK1 are similar in the blood stages, with maximum expression at the mature schizont stage (see Fig. S4 in the supplemental material). Based on these observations, we tested the effect of BKIs 1294 and 1613 on the kinase activity of recombinant *Pf*PKG. Interestingly, BKI 1294 (but not BKI 1613) inhibited the activity of recombinant *Pf*PKG. This result suggests that the sensitivity of CDPK1 T145M parasites to BKI 1294 could be due to its effect on *Pf*PKG, which directly or indirectly compensates the reduced activity of *Pf*CDPK1 in the mutant parasites. It could be possible that BKI 1613 affects the activity of an upstream kinase that masks its differential inhibition of CDPK1 in the CDPK1 T145M and wild-type parasites. We chose to test compound 2 (C2), a potent and specific inhibitor of *Pf*PKG, on the growth of CDPK1 T145M and wild-type parasites.

Treatment of wild-type parasites at the mature schizont stage with C2 led to arrest of the merozoites in the schizonts (22, 34). However, the T618Q mutation at the gatekeeper position of *Pf*PKG provided resistance in the *Pf*PKG T618Q transgenic parasites against the action of C2 (22, 34). These results validated *Pf*PKG as the primary target of C2. We tested different concentrations of C2 on the CDPK1 T145M and wild-type parasites. Interestingly, C2 had more inhibition of growth of CDPK1 T145M parasites than the wild type in the SYBR green I assay (Fig. 6B). The higher sensitivity of C2 for CDPK1 T145M parasites led to fewer of the ring-infected RBCs in the C2-treated mature schizonts compared to the wild type, indicating a block in egress or invasion. To partially address whether the effect is due to greater inhibition of egress or invasion, we counted the number of mature unruptured schizonts in the CDPK1 T145M and WT parasites under 300 nM C2 treatment. We focused on 300 nM C2-treated parasites since there was a striking difference in the CDPK1 T145M and the WT parasites with this concentration. The number of unruptured schizonts and the number of merozoites per unruptured schizont were similar in the CDPK1 T145M and the WT parasites under the 300 nM C2 condition. These results suggest that the increased sensitivity of the CDPK1 T145M parasites compared to the WT could be due to greater inhibition of invasion of RBCs in the CDPK1 T145M parasites with C2. Taken together, these results suggest that the reduced function of CDPK1 in the CDPK1 T145M parasites is compensated either directly by PKG or by other kinases under the control of PKG.

The levels of PKG transcript were similar in the wild-type and the CDPK1 T145M parasites (Fig. 7). Also, the transcript levels of CDPK4, a nonessential gene for asexual proliferation of the parasite, did not change significantly in the two parasites. Interestingly, CDPK5 and CDPK6 transcript expression was 1.43- and 1.23-fold, respectively, increased in the CDPK1 T145M parasites over the wild type. CDPK5 has been demonstrated to play a critical role in egress of merozoites from mature schizonts (36). It has been suggested that CDPK5 controls the final stages of parasite egress and that its function is downstream of *Pf*PKG. It could be possible that with reduced activity of CDPK1 in the CDPK1 T145M parasites, another kinase may synergize with CDPK1 during the invasion of RBCs. Taken together, the CDPK1 T145M parasites are more dependent on an alternate pathway directly controlled by PKG and likely involving other kinases. Moreover, upregulation and downregulation of other kinases (Fig. 7) suggest involvement of CDPK1 in other physiological processes in the parasite. It will be interesting to compare the global changes in the transcriptome or phosphoproteome of the CDPK1 T145M and the wild-type parasites that may give more insight into the compensatory mechanisms.

## MATERIALS AND METHODS

### Molecular cloning, expression, and purification of recombinant *Pf*CDPK1.

The coding sequence of the full-length *Pf*CDPK1 gene (PlasmoDB accession no. PF3D7_0217500) was amplified using the oligonucleotide pair pk1fpgex (5′ ATGCGCGGATCCATGGGGTGTTCACAAAGTTCAAACG 3′) and pk1rpgex (5′ ATGCGCGCGGCCGCTTATGAAGATTTATTATCACAAATTTTGTGCATC 3′) and cloned into pGEX4T1 expression vector (GE Healthcare Life Sciences, Piscataway, NJ) using BamHI and NotI restriction endonucleases (restriction sites underlined). The sequence-verified plasmid construct was transformed into the BLR(DE3)pLysS strain of *Escherichia coli*. *Pf*CDPK1 was expressed as a chimeric protein with glutathione *S*-transferase (GST) at the N terminus. *E. coli* cells were grown at 37°C until reaching an optical density (measured at 600 nm) of 0.7 to 0.9 followed by induction of protein expression with 1 mM isopropyl-β-d-1-thiogalactopyranoside (IPTG) for 5 h at 30°C. The *E. coli* cells were pelleted at 5,000 × *g* in a Sorvall RC6 Plus centrifuge and sonicated in buffer of the following composition: 1 mM dithiothreitol (DTT), 0.1 mg/ml lysozyme, 1 mM EDTA, 1 mM phenylmethylsulfonyl fluoride (PMSF), and a 1× protease inhibitor cocktail (Roche Life Science, Indianapolis, IN) in phosphate-buffered saline (PBS). The lysate was centrifuged at 17,000 × *g* for 1 h, and the clear supernatant with recombinant *Pf*CDPK1 was incubated overnight at 4°C with glutathione HiCap matrix (Qiagen, Valencia, CA). The glutathione HiCap matrix was extensively washed with PBS containing 1 mM DTT followed by a prewash with 50 mM Tris, 100 mM NaCl, 1 mM DTT, pH 7.5. The bound *Pf*CDPK1 was eluted with 25 mM reduced l-glutathione in 50 mM Tris, 100 mM NaCl, pH 7.5.

### Site-directed mutagenesis.

The following primer pairs were used to generate gatekeeper mutant constructs of *Pf*CDPK1 in a pGEX4T1 expression vector using the QuikChange II XL site-directed mutagenesis kit (Agilent Technologies, Santa Clara, CA) according to the manufacturer’s instructions: Ck1T145G (5′-GTTTGATGTTTTTGAAGATAAGAAATATTTTTATTTAGTAGGCGAATTTTATGAAGGTGGGGAA-3′) and Ck1T145G_antisense (5′-TTCCCCACCTTCATAAAATTCGCCTACTAAATAAAAATATTTCTTATCTTCAAAAACATCAAAC-3′), Ck1T145A (5′-GTTTGATGTTTTTGAAGATAAGAAATATTTTTATTTAGTAGCCGAATTTTATGAAGGTGGGGAA-3′) and Ck1T145A_antisense (5′-TTCCCCACCTTCATAAAATTCGGCTACTAAATAAAAATATTTCTTATCTTCAAAAACATCAAAC-3′), Ck1T145S (5′-GTTTGATGTTTTTGAAGATAAGAAATATTTTTATTTAGTAAGCGAATTTTATGAAGGTGGGGAA-3′) and Ck1T145S_antisense (5′-TTCCCCACCTTCATAAAATTCGCTTACTAAATAAAAATATTTCTTATCTTCAAAAACATCAAAC-3′), Ck1T145M (5′-GTTTGATGTTTTTGAAGATAAGAAATATTTTTATTTAGTAATGGAATTTTATGAAGGTGGGGAA-3′) and Ck1T145M_antisense (5′-TTCCCCACCTTCATAAAATTCCATTACTAAATAAAAATATTTCTTATCTTCAAAAACATCAAAC-3′), and Ck1T145Y (5′-GTTTGATGTTTTTGAAGATAAGAAATATTTTTATTTAGTATACGAATTTTATGAAGGTGGGGAA-3′) and Ck1T145Y_antisense (5′-TTCCCCACCTTCATAAAATTCGTATACTAAATAAAAATATTTCTTATCTTCAAAAACATCAAAC-3′). The final sequence-verified plasmids were transformed in the BLR(DE3)pLysS strain of *E. coli*. The gatekeeper mutant recombinant *Pf*CDPK1 proteins were expressed and purified as described above for the wild-type *Pf*CDPK1.

### *Pf*CDPK1 *in vitro* kinase activity assay.

The activities of the recombinant full-length *Pf*CDPK1 WT and gatekeeper mutants were evaluated using a semisynthetic approach as described by Allen et al. (14). Briefly, 50 ng of recombinant protein was incubated in buffer with the composition 50 mM Tris, 50 mM MgCl_2_, 1× phosphatase inhibitor cocktail (Roche Life Science, Indianapolis, IN) with or without a substrate, histone H3 (Cayman Chemical, Ann Arbor, MI). Conditions requiring the presence of calcium, 2.5 mM CaCl_2_, or absence, 2.5 mM EGTA (with no added calcium), were included in the buffer. Importantly, 100 μM ATPγS was used as the source of the transferable phosphate group. Additionally, two bulky ATP analogs, *N*_6_-benzyladenosine-5′-*O*-(3-thiotriphosphate), 6BnATPγS, and *N*_6_-furfuryladenosine-5′-*O*-(3-thiotriphosphate), 6FuATPγS (Axxora, Farmingdale, NY), were also used in the assays to evaluate the potential of the recombinant kinases for phosphorylation. The reaction mixture was incubated at 30°C for 1 h followed by terminating the reaction by addition of 5 mM EGTA. A 5 mM concentration of *p*-nitrobenzyl mesylate (PNBM) (Abcam, Inc., Cambridge, MA) was added into the reaction mixture and incubated at 20°C for 2 h to allow the alkylation of the thiophosphorylated serine or threonine residues. The reaction was stopped by addition of 1× LDS sample buffer (Thermo Fisher Scientific, Grand Island, NY).

### *Pf*PKG *in vitro* kinase activity assay.

Compound efficacy against recombinant *P. falciparum* cGMP-dependent protein kinase (*Pf*PKG) was determined using a novel nonradioactive and high-throughput assay. This assay evaluates protein kinase activity by measuring changes in initial ATP concentration via luminescence after *Pf*PKG enzyme phosphorylation of a peptide substrate, PKCtide (ERMRPRKRQGSVRRRV) (SignalChem, Richmond, BC, Canada). The *in vitro* kinase assay was set up with 26 µM PKCtide, 30 nM *Pf*PKG, and 20 µM cGMP in a buffered solution containing 20 mM β-glycerol phosphate, 20 mM MgCl_2_, 25 mM HEPES (pH 7.5) (KOH), 0.1% bovine serum albumin (BSA), and 2 mM DTT (37). The reaction was initiated with addition of 2 µM ATP. The reaction mixture was incubated at 30°C and 90-rpm agitation for 2 h. Reaction wells with no *Pf*PKG, no peptide substrate, and no cGMP were included in each assay plate as internal negative controls.

### Western blot analysis.

Western blot analysis was performed to detect the thiophosphorylated products of the *in vitro* kinase reaction. Briefly, the samples in 1× LDS sample buffer were separated by 4 to 12% PAGE followed by transfer to the polyvinylidene difluoride (PVDF) membrane. The nonspecific sites on the PVDF membrane were blocked by incubating it with 5% skim milk-0.1% Tween 20 in Tris-buffered saline (TBS; KD Medical, Columbia, MD) followed by incubation with a rabbit primary antibody, ab92570 (Abcam, Inc., Cambridge, MA), at a 1:2,500 dilution for 1 h at room temperature in the blocking buffer that binds specifically with the thiophosphorylated products. The blot was washed 3 times with 0.1% Tween 20 in TBS for 5 min each. The blot was further incubated with goat anti-rabbit secondary antibody, A6154 (Sigma-Aldrich, St. Louis, MO), in blocking buffer at a 1:5,000 dilution for 1 h at room temperature followed by washing with 0.1% Tween 20 in TBS. The blot was treated with SuperSignal West Pico chemiluminescent substrate (Thermo Fisher Scientific, Grand Island, NY) according to the manufacturer’s instructions and exposed on HyBlot CL autoradiography film. The film was developed on a Kodak X-Omat 2000A processor. Quantification of the thiophosphorylated protein bands was done using ImageJ software (version 1.49; National Institutes of Health [http://imagej.nih.gov/ij/]).

### BKI library screening.

Recombinant *Pf*CDPK1 WT and *Pf*CDPK1 T145M were used in the *in vitro* kinase assay to calculate the IC_50_s for the library of bumped kinase inhibitors. Thirty-one BKIs belonging to the pyrazolopyrimidine scaffold were screened (18–21). Protein kinase activity of recombinant enzymes was assayed using 20 µM synthetic peptide substrate PLARTLSVAGLPGKK (American Peptide Company, Inc., Sunnyvale, CA), which has been previously used as a substrate for *Pf*CDPK1 in an *in vitro* kinase assay (10); 5.516 nM *Pf*CDPK1; or 8.61095 nM *Pf*CDPK1 T145M. These reactions were performed in a buffered solution containing 1 mM EGTA (pH 7.2), 10 mM MgCl_2_, 20 mM HEPES (pH 7.5), 0.1% BSA, and enzyme activation reagent containing 2 mM CaCl_2_. The reactions were initiated with addition of 10 µM ATP, and the reaction mixtures were incubated for 90 min at 30°C. Inhibitory concentrations that give a 50% reduction in enzyme activity (IC_50_s) were determined over 8-point curves obtained in 96-well plates with compound dilutions from 2 µM to 0.0001 µM. The KinaseGlo (Promega, Madison, WI) luminescence-based assay, in which luminescence is inversely related to kinase activity and directly related to ATP depletion, was used as previously described (21).

### Construction of transfection plasmid constructs.

The 20-nucleotide guide sequence (5′ AACCGAATTTTATGAAGGTG 3′) targeting *Pf*CDPK1 (PF3D7_0217500) was selected by manual curation including the triplet coding for the gatekeeper residue (Fig. 5). The guide sequence was cloned using In-Fusion (Clontech, Mountain View, CA) into the pL6eGFP plasmid, giving rise to the pL6CK1Guide plasmid. The homology arm of 421 nucleotides (corresponding to nucleotides 133 to 553 of *Pf*CDPK1), specific for introducing a Met or Ser gatekeeper mutation, was cloned between SpeI and AflII in the pL6CK1Guide plasmid, making the final construct pL6CK1Met or pL6CK1Ser, respectively. The protospacer-adjacent motif (PAM) was mutated, and the target sequence in the homology arm was also changed to avoid repeated cleavage of the modified locus by Cas9 endonuclease after the desired editing (Fig. 5). For the expression of Cas9 endonuclease, the plasmid pUF1 was used as described by Ghorbal et al. (15).

### *In vitro* culture of *Plasmodium falciparum* NF54 and transfections.

The NF54 strain of *P. falciparum* was propagated *in vitro* in O^+^ human red blood cells (Virginia Blood Services, Richmond, VA) purified using a Sepacell R-500 II leukocyte reduction filter (Fenwal, Lake Zurich, IL) at 2% hematocrit in RPMI 1640 medium with l-glutamine, 25 mM HEPES, and 50 µg/ml hypoxanthine (KD Medical, Columbia, MD) supplemented with 0.5% AlbuMAX I (Thermo Fisher Scientific, Grand Island, NY) and 10 µg/ml gentamicin (Gibco, Thermo Fisher Scientific, Grand Island, NY) as described by Trager and Jensen (38). The parasites were electroporated as described earlier (39). Briefly, the ring-stage parasites at ~5% parasitemia were electroporated with 50 μg each of pL6CK1Met or pL6CK1Ser plasmid and pUF1 plasmid with the settings of 0.31 kV, 960 μF, and maximum capacitance on a Bio-Rad Gene Pulser II. WR99210 and DSM267 (40) were applied at the concentrations of 2 and 150 nM, respectively, after 24 h of transfection. The transgenic *Pf*CDPK1 T145M parasites were cloned by limiting dilution in a 96-well plate. The *Pf*CDPK1 gene sequence was amplified using primers ck1f1 (5′ ATTTTCTTTTCTGAACGTGTAACATG 3′) and ck1r3wt (5′ TGCATCTCTTAATCTCTCCTCACTG 3′) specific for the region outside the homology arm of the plasmid, and the desired mutation was confirmed by DNA sequencing.

### Parasite growth rate measurement.

Asexual parasites were synchronized for two consecutive cycles by sorbitol treatment (41) and set up at 0.2% trophozoite stage (30 to 36 h postinvasion) in a 96-well plate. The growth progression was monitored for 7 days, and the growth rate of the parasites was calculated by sampling the culture every 24 h and measuring the fluorescence readout using the SYBR green I assay as described earlier (42, 43).

### RT-PCR.

Eleven different protein kinase genes were manually selected to compare the transcript expression between the WT and CDPK1 T145M parasites. Primer pairs (listed in Table 4) were designed to amplify approximately 120 bp for each gene with similar melting temperatures using the OligoAnalyzer 3.1 tool from Integrated DNA Technologies, Inc. Parasites were harvested at 44 to 48 h postinvasion, and RNA was isolated using the RNeasy minikit (Qiagen, Valencia, CA). The RNA-containing samples were treated with the Turbo DNA-free kit (Thermo Fisher Scientific, Grand Island, NY) to remove contaminating DNA. Purified RNA was used to prepare cDNA using SuperScript III First-Strand Synthesis SuperMix (Thermo Fisher Scientific, Grand Island, NY) according to the manufacturer’s instructions. Target genes were amplified using iQ SYBR Green SuperMix (Bio-Rad Laboratories, Hercules, CA) and run on a Bio-Rad CFX Connect real-time system. The level of expression was normalized with two housekeeping genes, glyceraldehyde-3-phosphate dehydrogenase (GAPDH) and threonine tRNA ligase (ThrRS). Four independent biological replicates were done in triplicate for the final analysis using R (version 3.3.1) (44).

**TABLE 4:** Details of oligonucleotides used for real-time PCR

Gene (accession no.)	Oligonucleotide name	Sequence (5′–3′)
CDPK1 (PF3D7_0217500)	CDPK1RTF	GGAAGAATTAGCAAATTTATTTGGTTTGACATC
	CDPK1RTR	ATGTTAACGAATTCATCAAAGTCAATCATGT
CDPK2 (PF3D7_0610600)	CDPK2RTF	GGAACAGGAGAATTTACAACGAC
	CDPK2RTR	TGTATACATAATAACACCACTAGACCAG
CDPK3 (PF3D7_0310100)	CDPK3RTF	CACGAAATATTGAGCATGGTAAAGAAGG
	CDPK3RTR	CAGCGTCCATTGTAAGACATCTTTTTATTAAATC
CDPK4 (PF3D7_0717500)	CDPK4RTF	ATACTTCTCTCAGGGTGCCC
	CDPK4RTR	CTTATCACTAATTTTTTTGAATTGTGGTAAATCG
CDPK5 (PF3D7_1337800)	CDPK5RTF	GGAGGTCGAAGATATGGATACGAATAG
	CDPK5RTR	TATCGGCTAACGTACTCTTTGTCG
CDPK6 (PF3D7_1122800)	CDPK6RTF	CCTCCCGTAGATAAGAATATATTATCTATCG
	CDPK6RTR	ATCTGCTTCAATAAATCCCAATACATTTGC
CDPK7 (PF3D7_1123100)	CDPK7RTF	AGTCCTAAAAAAGATATATAAAGAACTAGGTAGTAG
	CDPK7RTR	TTTAAAAATAATCTTTCTCCCCACAACCC
PKA (PF3D7_0934800)	PKARTF	AATCATCCATTTTGTGTAAATTTACATGG
	PKARTR	CTTTTGTTTCTTCTTAAAAATGTAAAAAATTCTCC
PKG (PF3D7_1436600)	PKGRTF	AAAGGGAATGAAAGAAATAAAAAGAAGGC
	PKGRTR	CATATCAATATCTTCTGAAAGCTTTTCCC
PKB (PF3D7_1246900)	PKBRTF	CACAATAGAAGAAATGATGTTCTTTTTTACG
	PKBRTR	GAGAGCGCAATTAGCCATATTG
PI3K (PF3D7_0515300)	PI3KRTF	CCCCTTCAATTTGTTTGTGAAACAG
	PI3KRTR	ATCACATTTGTTATACTTATTATCATCACATTTGTT
GAPDH (PF3D7_1462800)	GAPDHRTF	GGAAGGAAAGATATCGAAGTAG
	GAPDHRTR	GGGTTACCTCACATGG
ThrRS (PF3D7_1126000)	TtRNALRTF	CTTGGGAACTGCAGAGTAGAATTT
	TtRNALRTR	TAAAAATCCTCCGAACAATTTTTCTAAACTAC

### Invasion experiments with C2.

*P. falciparum* schizont-stage parasites were harvested at 44 to 48 h postinvasion using a Percoll gradient. The final parasitemia was adjusted to 2% with uninfected RBCs at 2% hematocrit. The parasites were treated with different concentrations of C2 for 8 h. The effect of C2 was evaluated by counting the number of ring-stage parasites in the blood smears stained with Giemsa stain under a light microscope. The percent ring parasitemia was plotted against different concentrations of C2. Two independent biological experiments done in triplicate were used for making the graph using GraphPad Prism 6. The images of the Giemsa-stained smears were acquired with Evos XL Core with a Zeiss 100× 1.45-numerical-aperture (NA) objective lens (Thermo Fisher Scientific, Grand Island, NY).

## SUPPLEMENTAL MATERIAL

Figure S1  Growth rates of wild-type and CDPK1 T145M parasites are similar. The growth curves of the wild type and two clones of CDPK1 T145M parasites (2 and 4) were generated using the SYBR green I fluorescence assay as described in Materials and Methods. The figure shows the plot of normalized fluorescence values against the number of days for the wild-type (black circles), CDPK1 T145M 2 (black squares), and CDPK1 T145M 4 (black triangles) parasites. The figure has been generated using data from two independent experiments done in triplicate. Download Figure S1, TIF file, 0.1 MB

Figure S2  Effect of bumped kinase inhibitors on asexual growth of wild-type and CDPK1 T145M parasites. The effect of bumped kinase inhibitors (BKIs) was evaluated on the asexual proliferation of wild-type and CDPK1 T145M parasites as described in Materials and Methods. The figure shows the effect of different concentrations of BKI 1613 on the growth of wild-type (blue) and CDPK1 T145M (red) parasites. The percent inhibition of parasite growth is plotted against different molar concentrations of BKI 1613 expressed as log_10_[1613]. BKI 1613 showed no difference in inhibition of the growth of the two parasites. The figure has been generated using data from two independent experiments done in duplicate with GraphPad Prism 6. Download Figure S2, TIF file, 0.1 MB

Figure S3  Effect of 300 nM compound 2 (C2) on CDPK1 T145M and wild-type parasites. (A) Numbers of merozoites were counted in the unruptured schizonts under 300 nM C2 treatment. The number of merozoites per unruptured schizont with 300 nM C2 is plotted for the WT and the CDPK1 T145M parasites. The graph was generated using GraphPad Prism 6. The error bars represent standard deviations in two independent experiments done in duplicate. (B) Giemsa-stained smears were used to acquire images of the CDPK1 T145M and WT parasites 8 h following the treatment with and without 300 nM C2. The figure shows the ring stage in the WT and the CDPK1 T145M parasites after 8 h without (a and b) or with (c, d, e, and f) 300 nM C2 treatment. The unruptured schizonts are shown for the WT and CDPK1 T145M parasites (e and f) after the 300 nM C2 treatment. Download Figure S3, TIF file, 0.7 MB

Figure S4  Transcript profile of CDPK1 and PKG in blood stages of *P. falciparum*. The transcript expression of CDPK1 and PKG was analyzed in synchronized ring, trophozoite, and schizont stages of *P. falciparum* by real-time PCR as described in Materials and Methods. The transcript level of CDPK1 and PKG increases progressively during the schizogony with maximum expression in the mature schizont stage (44 to 48 h postinvasion of a 48-h cycle). The graph shows the fold change in the transcript expression of CDPK1 and PKG across rings, trophozoites, and schizonts. The figure has been generated using two independent experiments performed in triplicate and drawn using GraphPad Prism 6. Download Figure S4, TIF file, 0.1 MB
