# Supplementary figures and images for: Reduced Activity of Mutant Calcium-Dependent Protein Kinase 1 Is Compensated in Plasmodium falciparum through the Action of Protein Kinase G
Source: mBio. 2016 Dec 6;7(6):e02011-16. doi: 10.1128/mBio.02011-16 (PMC5142624; doi:10.1128/mBio.02011-16)

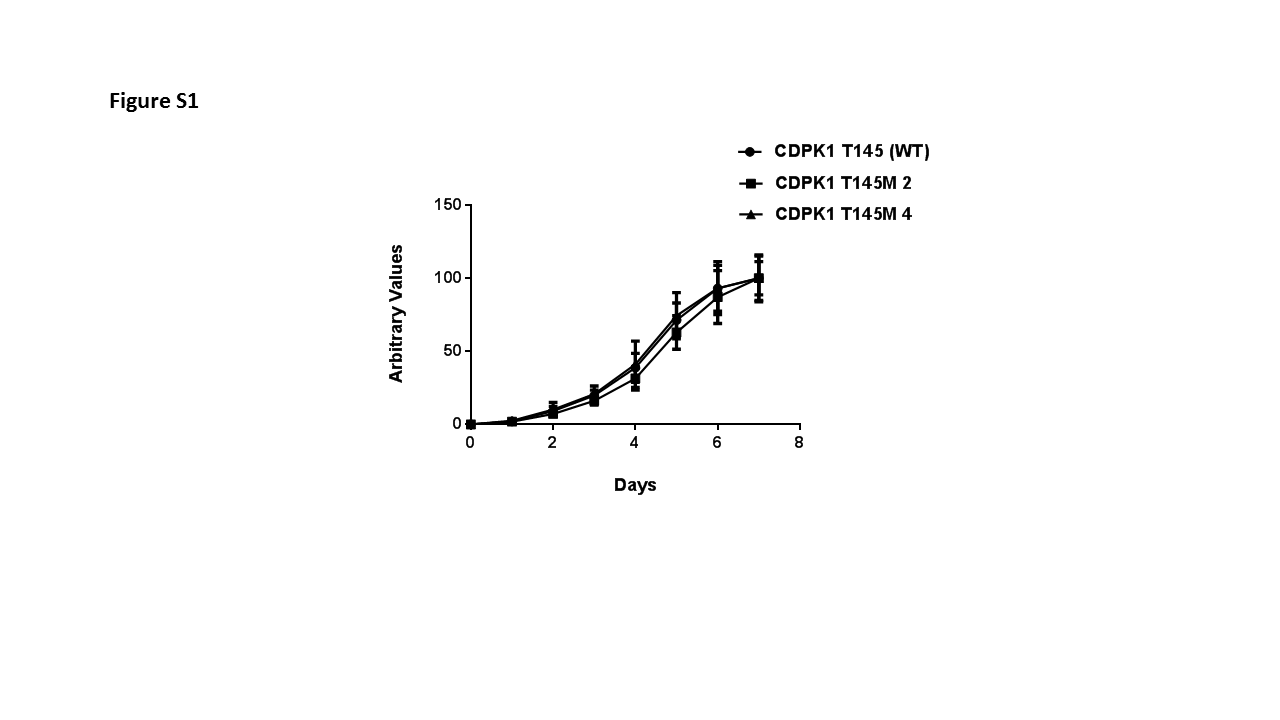

Supplement: Figure S1 — Growth rates of wild-type and CDPK1 T145M parasites are similar. The growth curves of the wild type and two clones of CDPK1 T145M parasites (2 and 4) were generated using the SYBR green I fluorescence assay as described in Materials and Methods. The figure shows the plot of normalized fluorescence values against the number of days for the wild-type (black circles), CDPK1 T145M 2 (black squares), and CDPK1 T145M 4 (black triangles) parasites. The figure has been generated using data from two independent experiments done in triplicate. Download [file mbo006163095sf1.tif]

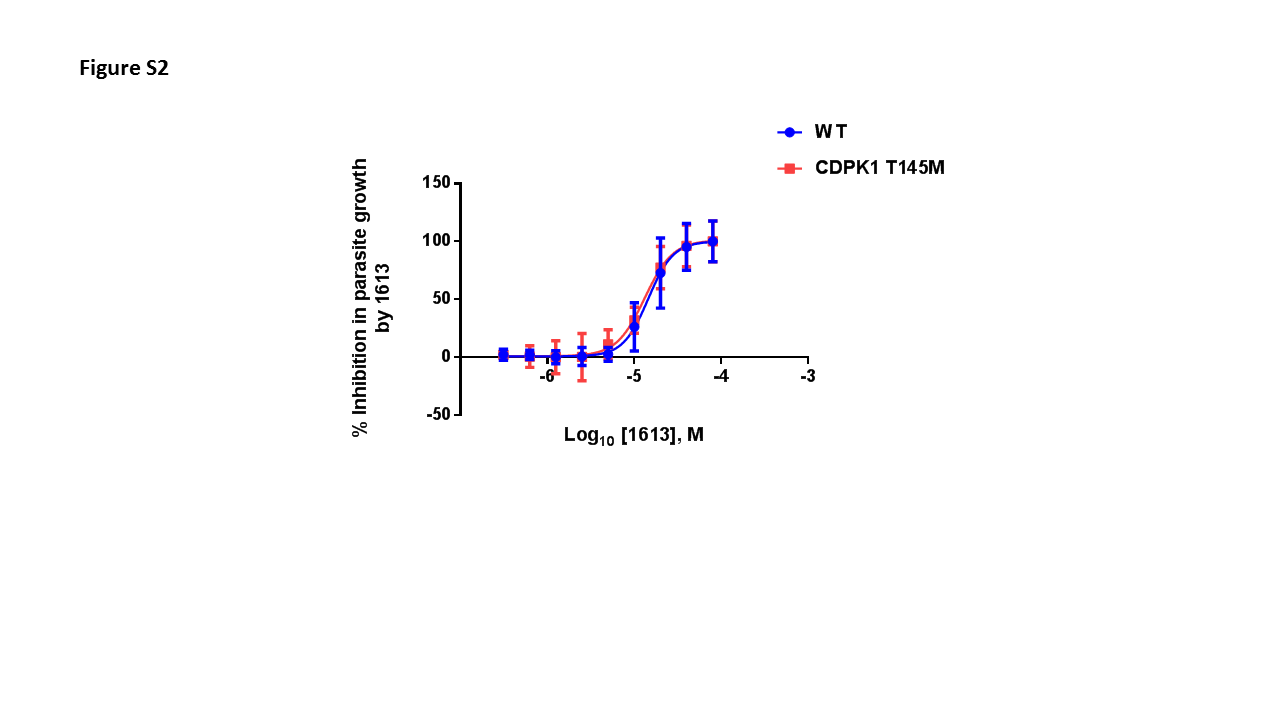

Supplement: Figure S2 — Effect of bumped kinase inhibitors on asexual growth of wild-type and CDPK1 T145M parasites. The effect of bumped kinase inhibitors (BKIs) was evaluated on the asexual proliferation of wild-type and CDPK1 T145M parasites as described in Materials and Methods. The figure shows the effect of different concentrations of BKI 1613 on the growth of wild-type (blue) and CDPK1 T145M (red) parasites. The percent inhibition of parasite growth is plotted against different molar concentrations of BKI 1613 expressed as log10[1613]. BKI 1613 showed no difference in inhibition of the growth of the two parasites. The figure has been generated using data from two independent experiments done in duplicate with GraphPad Prism 6. Download [file mbo006163095sf2.tif]

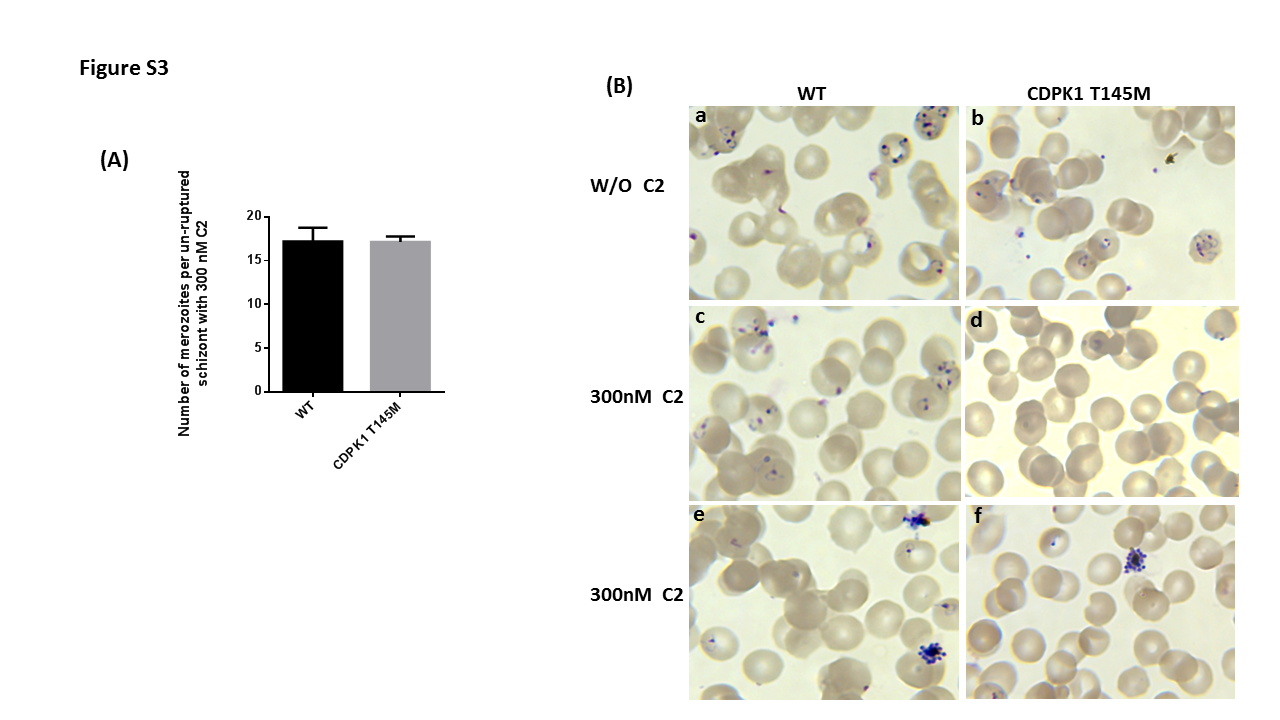

Supplement: Figure S3 — Effect of 300 nM compound 2 (C2) on CDPK1 T145M and wild-type parasites. (A) Numbers of merozoites were counted in the unruptured schizonts under 300 nM C2 treatment. The number of merozoites per unruptured schizont with 300 nM C2 is plotted for the WT and the CDPK1 T145M parasites. The graph was generated using GraphPad Prism 6. The error bars represent standard deviations in two independent experiments done in duplicate. (B) Giemsa-stained smears were used to acquire images of the CDPK1 T145M and WT parasites 8 h following the treatment with and without 300 nM C2. The figure shows the ring stage in the WT and the CDPK1 T145M parasites after 8 h without (a and b) or with (c, d, e, and f) 300 nM C2 treatment. The unruptured schizonts are shown for the WT and CDPK1 T145M parasites (e and f) after the 300 nM C2 treatment. Download [file mbo006163095sf3.tif]

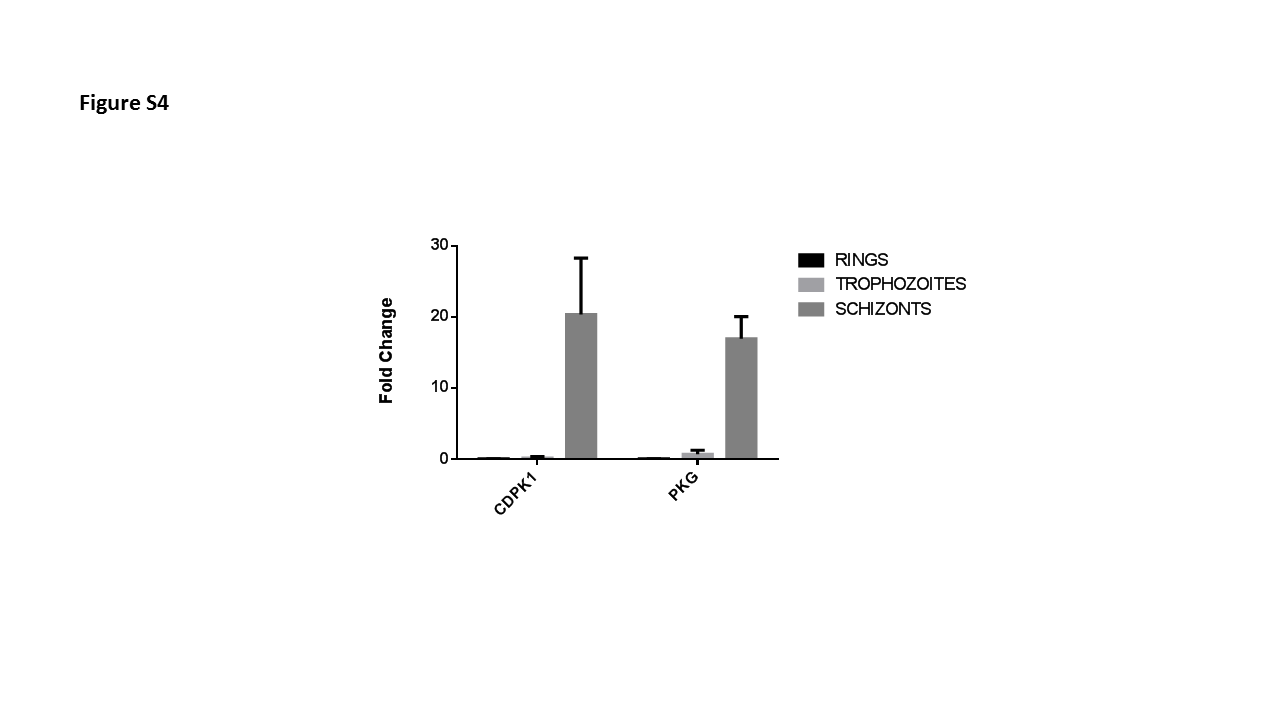

Supplement: Figure S4 — Transcript profile of CDPK1 and PKG in blood stages of P. falciparum. The transcript expression of CDPK1 and PKG was analyzed in synchronized ring, trophozoite, and schizont stages of P. falciparum by real-time PCR as described in Materials and Methods. The transcript level of CDPK1 and PKG increases progressively during the schizogony with maximum expression in the mature schizont stage (44 to 48 h postinvasion of a 48-h cycle). The graph shows the fold change in the transcript expression of CDPK1 and PKG across rings, trophozoites, and schizonts. The figure has been generated using two independent experiments performed in triplicate and drawn using GraphPad Prism 6. Download [file mbo006163095sf4.tif]
